# Supplementary material for: Comparison of growth texture in round Bi2212 and flat Bi2223 wires and its relation to high critical current density development
Source: Sci Rep. 2015 Feb 10;5:8285. doi: 10.1038/srep08285 (PMC4322349; doi:10.1038/srep08285)
Supplement: Supplementary Information [file srep08285-s1.pdf]

Supplementary figures for;

Comparison of growth texture in round Bi2212 and flat Bi2223 wires and its relation to high critical current density development

F. Kametani, J. Jiang, M. Matras, D. Abraimov, E. E. Hellstrom and D. C. Larbalestier

Applied Superconductivity Center, National High Magnetic Field Laboratory, Florida State University, Tallahassee FL 32310 USA

Figure captions

Figure S1 600 µm long ND-IPF cross section montage of the Bi2212 filament that is split into 5 images. The images are continuous according to the numbering order. The right end of one image connects to the left end of the following image.

Figure S2 The ND-IPF map of a longitudinal cross section of Bi2212 filament. The two locations on the single Bi2212 grain are denoted as A and B, for which the schematic unit cells are shown to illustrate the crystallographic orientations. From the location A to B, the Bi2212 ab-planes twist ~30° around the wire direction, and bend ~5° upward by plastic deformation that occurs during grain formation and growth at high temperature.

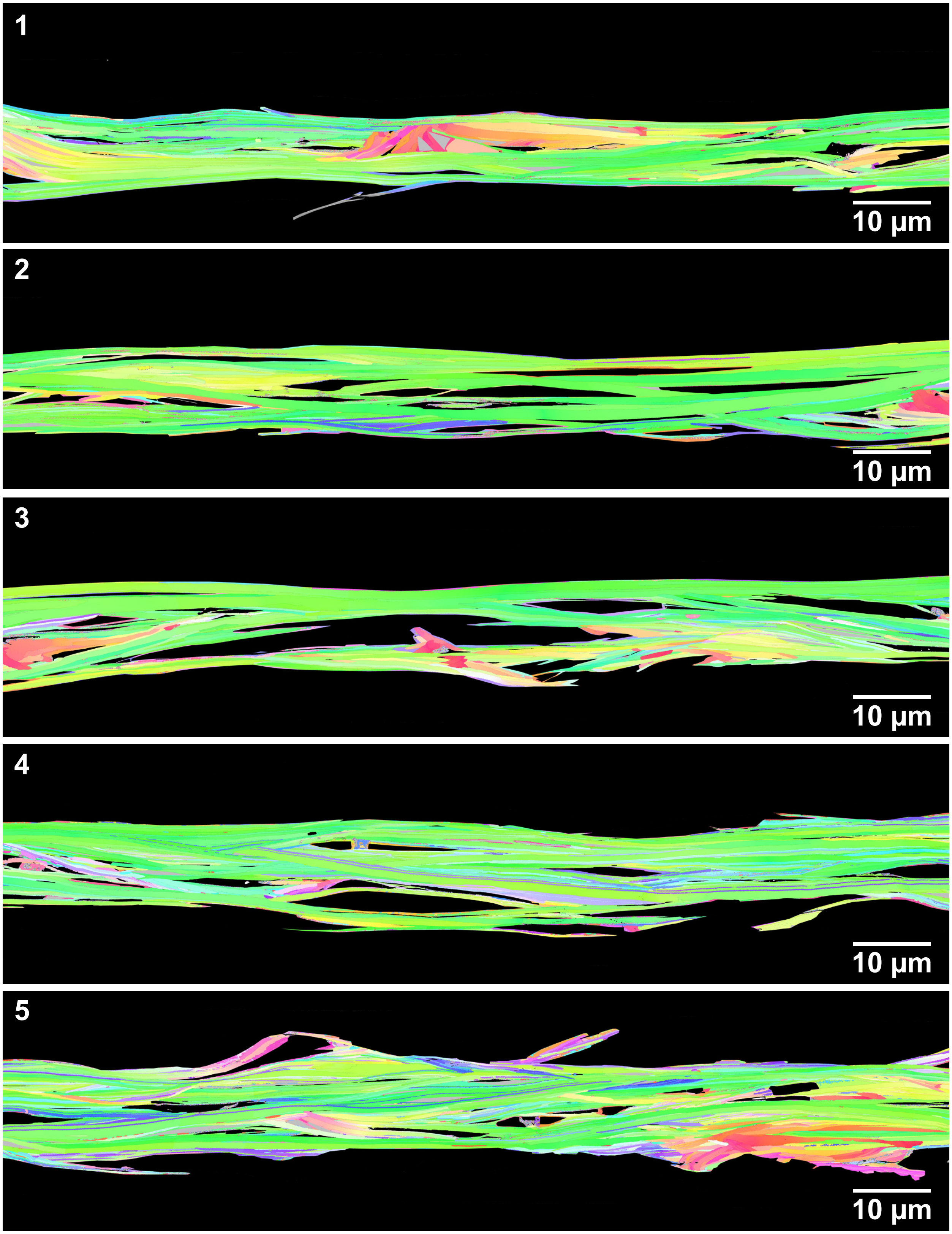

Figure S1

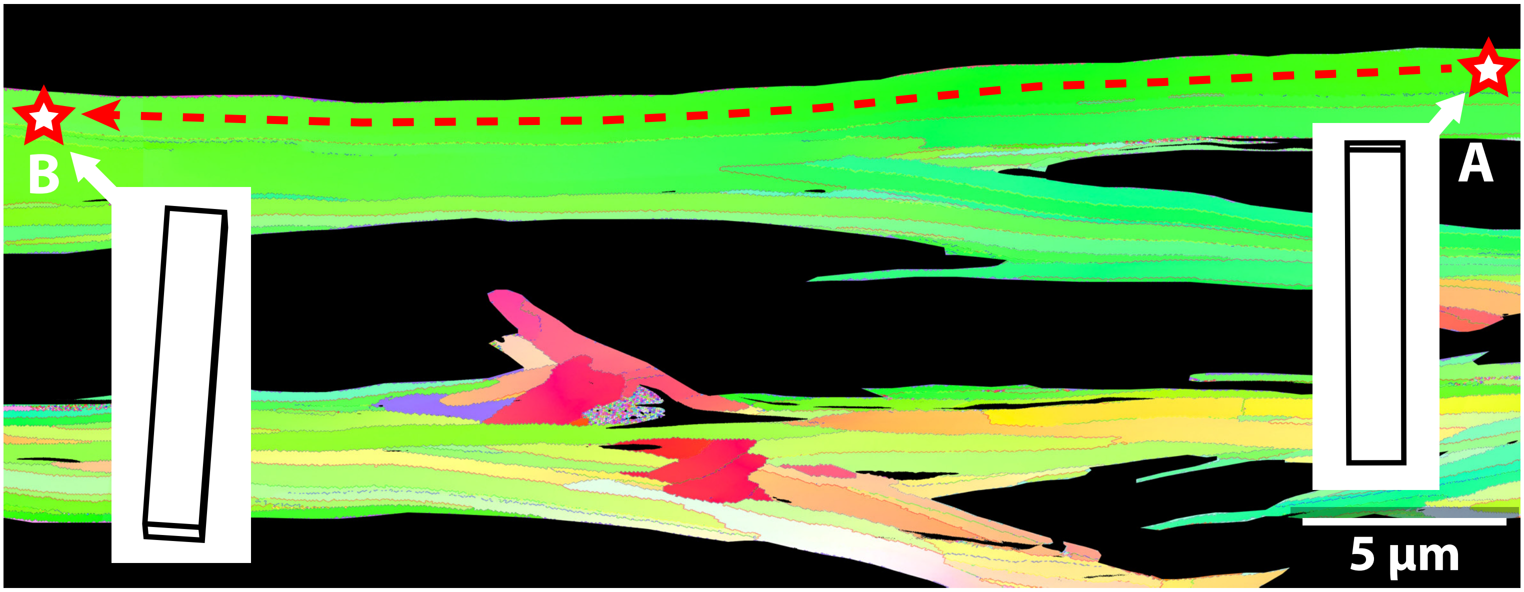

Figure S2
